# Enhanced Glycogen Storage of a Subcellular Hot Spot in Human Skeletal Muscle during Early Recovery from Eccentric Contractions

**DOI:** 10.1371/journal.pone.0127808

**Published:** 2015-05-21

**Authors:** Joachim Nielsen, Jean Farup, Stine Klejs Rahbek, Frank Vincenzo de Paoli, Kristian Vissing

**Affiliations:** 1 Department of Sports Science and Clinical Biomechanics, SDU Muscle Research Cluster (SMRC), University of Southern Denmark, Odense M, Denmark; 2 Department of Pathology, SDU Muscle Research Cluster (SMRC), Odense University Hospital, Odense C, Denmark; 3 Section of Sport Science, Department of Public Health, Aarhus University, Aarhus, Denmark; 4 Department of Biomedicine, Aarhus University, Aarhus, Denmark; Tohoku University, JAPAN

## Abstract

Unaccustomed eccentric exercise is accompanied by muscle damage and impaired glucose uptake and glycogen synthesis during subsequent recovery. Recently, it was shown that the role and regulation of glycogen in skeletal muscle are dependent on its subcellular localization, and that glycogen synthesis, as described by the product of glycogen particle size and number, is dependent on the time course of recovery after exercise and carbohydrate availability. In the present study, we investigated the subcellular distribution of glycogen in fibers with high (type I) and low (type II) mitochondrial content during post-exercise recovery from eccentric contractions. Analysis was completed on five male subjects performing an exercise bout consisting of 15 x 10 maximal eccentric contractions. Carbohydrate-rich drinks were subsequently ingested throughout a 48 h recovery period and muscle biopsies for analysis included time points 3, 24 and 48 h post exercise from the exercising leg, whereas biopsies corresponding to prior to and at 48 h after the exercise bout were collected from the non-exercising, control leg. Quantitative imaging by transmission electron microscopy revealed an early (post 3 and 24 h) enhanced storage of intramyofibrillar glycogen (defined as glycogen particles located within the myofibrils) of type I fibers, which was associated with an increase in the number of particles. In contrast, late in recovery (post 48 h), intermyofibrillar, intramyofibrillar and subsarcolemmal glycogen in both type I and II fibers were lower in the exercise leg compared with the control leg, and this was associated with a smaller size of the glycogen particles. We conclude that in the carbohydrate-supplemented state, the effect of eccentric contractions on glycogen metabolism depends on the subcellular localization, muscle fiber’s oxidative capacity, and the time course of recovery. The early enhanced storage of intramyofibrillar glycogen after the eccentric contractions may entail important implications for muscle function and fatigue resistance.

## Introduction

In addition to muscle damage, muscle soreness and transient muscle force loss [1,2], unaccustomed eccentric exercise also affects muscle metabolism [3]. In particular, glycogen synthesis is impaired after muscle-damaging eccentric contractions [4–7] and has been associated with reductions in GLUT 4 content and translocation [7] as well as reduced glucose uptake [8,9].

Recently, the role and regulation of muscle glycogen have been specified to be dependent on its subcellular localization [10]. This is based on pioneering studies using transmission electron microscopy conducted in the 1970s and 1980s showing both fiber type differences and a localization-dependent utilization of glycogen during exercise [11–15]. Later, by a quantitative approach, three subcellular locations of glycogen have been defined [16]: 1) Intermyofibrillar glycogen where glycogen particles are located between the myofibrils next to sarcoplasmic reticulum and mitochondria; 2) Intramyofibrillar glycogen, which is glycogen particles located within the myofibrils between the contractile filaments; and 3) Subsarcolemmal glycogen defined as the glycogen particles situated from the outermost myofibril to the surface membrane.

Interestingly, two recent studies suggest that intramyofibrillar glycogen may be affected by muscle protein degradation. This is deduced from the finding that 2 weeks of immobilization induced a loss of 50% of the glycogen particles located in the intramyofibrillar region, whereas intermyofibrillar and subsarcolemmal regions of glycogen deposition were unaffected [17]. Moreover, another study showed that resynthesis of intramyofibrillar glycogen, as judged by glycogen particle number, was impaired during the second day of recovery from a soccer match compared with the other depositions of glycogen [18]. This was observed despite the players received a high-carbohydrate (and high in creatine) diet and is in contrast to the preferential resynthesis of intramyofibrillar glycogen observed after glycogen-depleting cycling exercise [19]. Thus, the slowed resynthesis of glycogen following eccentric exercise is not due to inadequate carbohydrate intake and seems mostly confined to intramyofibrillar glycogen.

Intriguingly, eccentric contractions are accompanied by focal disruption of myofibrils mostly at the level of Z-disks [20] suggesting that glycogen particles located within the myofibrils could be more affected by eccentric contractions than glycogen particles located distant to the myofibrils (intermyofibrillar and subsarcolemmal depositions, respectively). In the above mentioned studies, no difference between high oxidative (type I) and low oxidative (type II) fibers were reported. Nonetheless, type II fibers are contended to be more susceptible to muscle damage [20–22], suggesting that changes in glycogen metabolism following muscle-damaging eccentric contractions may also occur in a fiber type-dependent manner.

Therefore, a better understanding of the effect of muscle-damaging eccentric contractions on glycogen metabolism necessitates analysis of fiber type-specific glycogen distribution between discrete subcellular localizations and analysis of the balance between the regulation of glycogen content via determination of particle size and number.

To investigate this, healthy human male subjects performed 15 sets of 10 repetitions of maximal isokinetic eccentric contractions for the knee extensors, as previously described [23]. During the 48h post-exercise recovery period with high-carbohydrate diet, muscle force produced with maximal voluntary contractions decreased, whereas serum creatine kinase levels increased and muscle pain was assessed as moderate using the visual analog scale [23], collectively indicating muscle damage and muscular fatigue. The specific aims of the present part of the study were; (1) to investigate the effect of muscle-damaging eccentric contractions on the subcellular distribution and content of glycogen during post-exercise recovery and; (2) if particle number or particle size of glycogen were affected in a fiber type-dependent manner. We hypothesized that the muscle-damaging eccentric contractions would preferentially mediate a decrease in the number of intramyofibrillar glycogen particles and that this would primarily adhere to type II fibers. In order to avoid any decrease in glycogen content due to inadequate carbohydrate intake, subjects received a diet high in carbohydrates during the entire recovery period.

## Materials and Methods

### Subjects

Five healthy young recreationally active men were included in this study, which was part of a larger study previously described in [23]. The five subjects were randomly selected from a larger cohort and characterized as follows; height (cm), age (years), body mass (kg) and body fat (%) were 184.8 (180–192) cm, 23.2 (21–25) years, 80.6 (70.6–92.9) kg, 15.3 (8.4–22.5) %, respectively. All subjects were informed of the purpose and risks of the study and provided written informed consent in accordance with the Declaration of Helsinki. The study was approved by the Central Denmark Region Committees on Health Research Ethics (ref. no. M-20110179).

### Experimental design

The study design and protocol has been described in detail previously [23]. In brief, a total of five needle biopsies obtained from the middle part of vastus lateralis muscle were included in this study: 2 weeks before experimental day from the non-exercising leg (pre), 3 h (post 3h), 24 h (post 24h) and 48 h (post 48h) after completion of an eccentric exercise protocol (see below). Furthermore, a biopsy was obtained from the non-exercising leg at 48 h post-exercise (control 48h). For all post exercise biopsies the sampling sites were attempted to cover a large area within the middle section of the vastus lateralis muscle to minimize any effects inflicted by the previous biopsies. Lidocaine (10 mg/ml) was used to anaesthetize the skin and subcutaneous tissue prior to the biopsies.

The biopsy from the control leg that was obtained 2 weeks prior to the single-bout exercise trial and was obtained in the morning after an overnight fast. On the exercise day subjects arrived to the laboratory also in a fasted condition and performed the eccentric exercise protocol, which consisted of 15 x 10 repetitions of maximal isokinetic eccentric contractions (30 dg/s, 70 dg range of motion) for the knee extensors in an isokinetic dynamometer (Humac Norm, CSMI, Stoughton, USA). Repetitions and sets were interspaced by 3 and 60 s, respectively. Immediately after completion of the exercise protocol (approximately 10am), subjects ingested a high carbohydrate-drink (952 kJ in an 8% solution consisting of 56 g of carbohydrate) before the post 3 h muscle biopsy was obtained (i.e. the CHO-drink was ingested 3 h before the 3 h recovery biopsy). The second drink was ingested before leaving the laboratory (1.00 pm) and the subjects received a third drink to ingest three hours later (4.00 pm). On day 1 and 2 following the exercise bout the subjects were instructed to ingest the carbohydrate drink at absolute time-points similar to the exercise day (i.e. 10 am, 1 pm and 4 pm). The 24 h and 48 h post exercise biopsies were all obtained following an overnight fast before carbohydrate drink consumption. Overall, before the 3, 24 and 48 h post-exercise biopsies subjects ingested 56, 168 and 168 g of CHO via the carbohydrate drinks, respectively. All subjects were carefully instructed to maintain their habitual diet during the study period and the dietary records obtained from the exercise day and day 1 and 2 post exercise revealed a normal energy intake and macronutrient distribution [23].

### Transmission electron microscopy and stereological procedure

Small segments (< 1 mm^3^) of the muscle biopsy samples were prepared for glycogen visualization by transmission electron microscopy as described previously [17]. Three sections interspaced by 150 μm were cut from each biopsy using an ultramicrotome (Leica Ultracut UCT ultramicrotome; Rowako AB, Vendelsö, Sweden). All the longitudinal oriented fibers (5 (5:6) median and IQR) were photographed by transmission electron microscopy (JEM-1400Plus, JEOL Ltd, Tokoyo, Japan) in a randomized systematic order including 12 images of x 10,000 magnification from both the subsarcolemmal and myofibrillar (superficial and central) regions [17]. In order to minimize any selection bias, the fibers were selected at low magnification (x800) based solely on the degree of longitudinal orientation as observed by the alignment of the myofibrils. Thus, the selection of fibers was unbiased by any ultrastructural appearance (i.e. z-disk streaming, glycogen or mitochondria). All the fibers were selected and photographed by JN. Since most studies suggest that all motor units are recruited during maximal voluntary eccentric contractions [24], we assume that all the photographed fibers were recruited during the exercise protocol of the present study.

### Muscle damage as evaluated by Z-disk streaming

**Fig 1:**
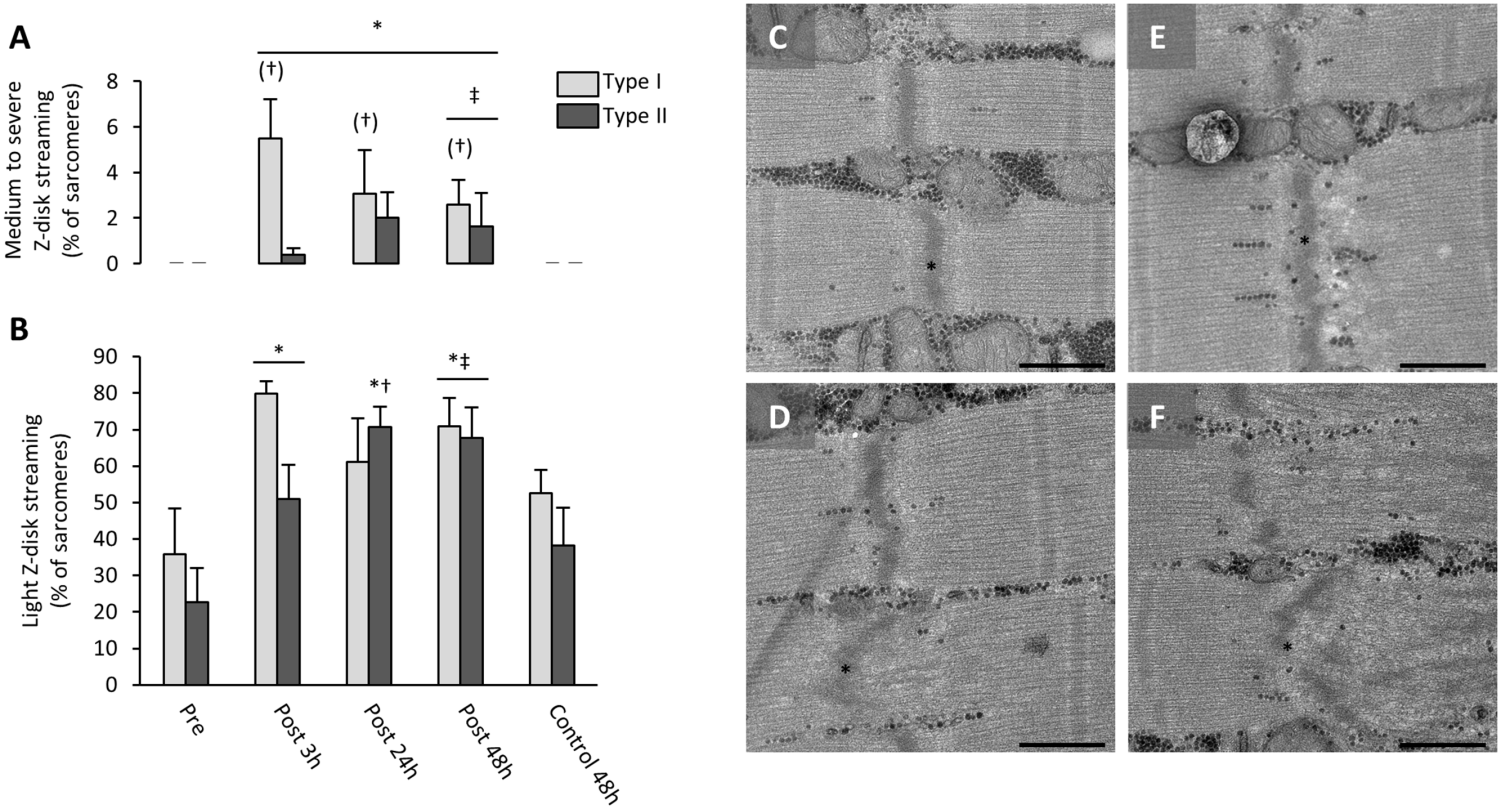
Increased muscle ultrastructural damage after eccentric contractions. Ultrastructural damage was evaluated based on Z-disk streaming as light damage or medium to severe damage in type I (light grey) and type II (dark grey) fibers. A) The percentage of sarcomeres showing medium to severe Z-disk damage. *: different from Pre (*P* = 0.02, Kruskal-Wallis test). (†): tendency to be different from type II fibers (*P* = 0.056, Kruskal-Wallis test). ‡: different from Con 48h (*P* = 0.008, Kruskal-Wallis test). B) The percentage of sarcomeres showing light Z-disk damage. *: different from Pre (*P* < 0.0001). #: different from type II fibers (*P* = 0.02). ‡: different from control 48 h (*P* = 0.0004). Values represent means and horizontal bars represent SEM. Representative transmission electron micrographs illustrates Z-disks (asterisk) with no streaming (C) and light (D), medium (D) and severe (E) Z-disk streaming. Scale bar = 0.5 μm.

Muscle damage was evaluated by assessing the degree of Z-disk streaming as described previously [20]. The degree of muscle damage was evaluated as the percentage of sarcomeres with light (slight irregular Z-disk), medium (Z-disk streaming into the I-band) or severe (Z-disk streaming into the A-band) Z-disk streaming (Fig 1C–1F). However, due to a low frequency of Z-disks with medium or severe streaming, the results for those two were combined.

### Subcellular glycogen localization

Glycogen volume in intermyofibrillar, intramyofibrillar and subsarcolemmal localizations were estimated by point counting as proposed by Weibel ([25], their eqn (4.20)) correcting for section thickness and glycogen particle diameter (see also [26]). Subsarcolemmal glycogen was estimated by point counting using a grid size of 135 nm and related to the surface area of the fiber as estimated by measuring the length of the fiber. The estimated coefficient of error (CE_est_) (see [27]) for subsarcolemmal glycogen was 0.19. Intermyofibrillar and intramyofibrillar glycogen were estimated by point counting using a grid size of 180 nm and 60 nm, respectively, and related to the myofibrillar and intramyofibrillar volume, respectively, as estimated by point counting using a grid size of 300 nm. In order to take into account the cylindrical shape of the fibers, where the superficial region has a higher fractional contribution of the cell volume than the central region, the images from the superficial and central regions were weighted 3:1. The CE_est_ for intermyofibrillar and intramyofibrillar glycogen volume were 0.14 and 0.15, respectively. All analyses were conducted by the same blinded investigator. Reproducibility tests showed no signs of bias and low coefficients of variation (*<* 6%) evaluated as proposed by Bland & Altman [28].

Glycogen particle diameter was directly measured using iTEM (FEI Company, Eindhoven, The Netherlands). The coefficient of variation between the sizes of particles reaches a constant level (approximately 16%) after measurement of 60–70 particles. Therefore, at least 70 particles were measured in each of the three locations (subsarcolemmal, intermyofibrillar and intramyofibrillar) per fiber.

As described above, intermyofibrillar and intramyofibrillar glycogen are expressed as volume densities, whereas subsarcolemmal glycogen is expressed as volume per fiber surface area. In order to assess the relative distribution of the different glycogen pools, subsarcolemmal glycogen is also expressed relative to the volume beneath the surface area of a cylindrically shaped fiber (*V*_b_) = *R*×0.5×*A*, where *R* is fiber radius and *A* is the fiber surface area. The fiber radius was assumed to be 40 μm.

### Mitochondrial content and fiber typing

Mitochondrial volume per fiber volume was estimated as described previously [17] using point counting with a grid size of 135 nm. The CE_est_ for mitochondria volume was 0.26. Fibers were classified as type I or II (IIa + IIb) based on mitochondrial content [29]. In order to minimize the number of incorrectly allocated fibers (see [29]), only fibers with mitochondrial content within the highest (n = 44) and lowest (n = 44) tertile were categorized as type I and II fibers, respectively, and the fibers (n = 43) with mitochondrial content within the middle tertile were not included in the analysis of fiber type differences in the degree of muscle damage and the glycogen parameters. The mean (± SD) mitochondrial volume percentage was 11.7 (2.1) % and 3.9 (1.3) % of type I and II fibers, respectively. The number of type I and II fibers at the various time points were 8 and 9 (pre), 8 and 9 (post 3 h), 8 and 9 (post 24 h), 11 and 9 (post 48 h), and 9 and 8 (control 48 h), respectively. The number of fibers per subject at the different time points is shown in S1 Fig.

### Statistics and data treatment

Statistical analyses were performed using STATA 13.1 (StataCorp. 2013. Stata Statistical Software: Release 13. College Station, TX: StataCorp LP). All interactions or main effects were tested using a linear mixed-effects model with subjects as random effects and with time, fiber type and location (glycogen parameters only) as fixed effects. Model assumptions about normal distribution of residuals and homogeneity of variance were satisfied by transformation of data. When model assumptions failed, the non-parametric Kruskal-Wallis test was used as indicated. Values are presented as geometric means and 95% confidence intervals, unless stated otherwise. Significance level was set at α = 0.05.

## Results

### Muscle damage

The muscle damage as evaluated by z-disk streaming is shown in Fig 1. Medium to severe z-disk streaming was only observed in the recovery period of the eccentric leg (Fig 1A), and there was a tendency to a higher proportion of damaged z-disks in type I fibers compared with type II fibers (Fig 1A). The light z-disk streaming was found to be dependent on fiber type and recovery period (P = 0.04) characterized by an increase in light z-disk streaming in both fiber types at 3 h and 48 h after the exercise, but most pronounced in type II fibers at 24 h after the exercise (Fig 1B).

### Subcellular glycogen distribution

**Fig 2:**
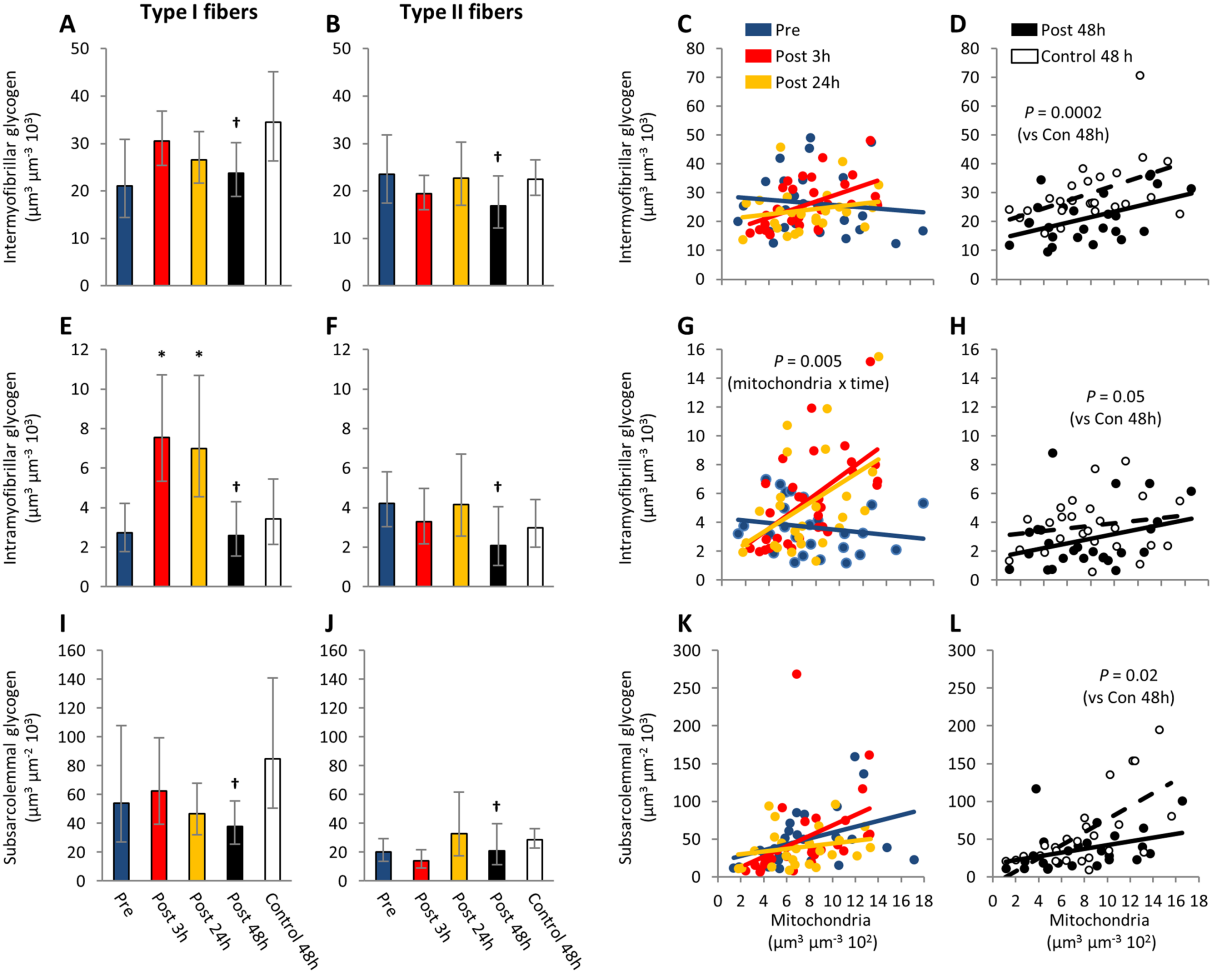
Enhanced intramyofibrillar glycogen storage early (3–24 h) during recovery from eccentric contractions but later (48 h) in recovery all subfractions of glycogen are lower in the exercise leg compared with the control leg. Intermyofibrillar (A-D), intramyofibrillar (E-F) and subsarcolemmal (I-L) glycogen content at the various time points are shown in type II fibers (A, E, I) and type I fibers (B, F, J). Bars represent geometric means and horizontal lines represent 95% confidence interval. Scatter plots of glycogen content against mitochondria content of individual fibers are shown for pre, post 3h and post 24h (C, G, K) and for post 48h and control 48h (D, H, L). Lines represent best-fitted linear regressions. †: different from control 48h (*P* = 0.00001). *: different from the other time points (*P* < 0.001).

There was a three-way interaction between subcellular location of glycogen, fiber type, and time course of recovery (*P* = 0.001). The eccentric contractions mediated an early (3 h post) increase by 150% (63:283) in intramyofibrillar glycogen within the type I fibers only (Fig 2E vs 2F). This elevated level of intramyofibrillar glycogen persisted at 24 h (168% (75:311) of pre exercise level), but not at 48 h, where it was not different from the pre exercise level (-7% (-38:39), P = 0.85). This enhanced storage of intramyofibrillar glycogen was not observed within the type II fibers (Fig 2E and 2G) or in intermyofibrillar (Fig 2A–2C) or subsarcolemmal (Fig 2I–2K) glycogen content. At 48 h post exercise the glycogen levels of all three locations were reduced by -35% (-46:-21) in the eccentric leg compared with the control leg in both fiber types (Fig 2).

**Fig 3:**
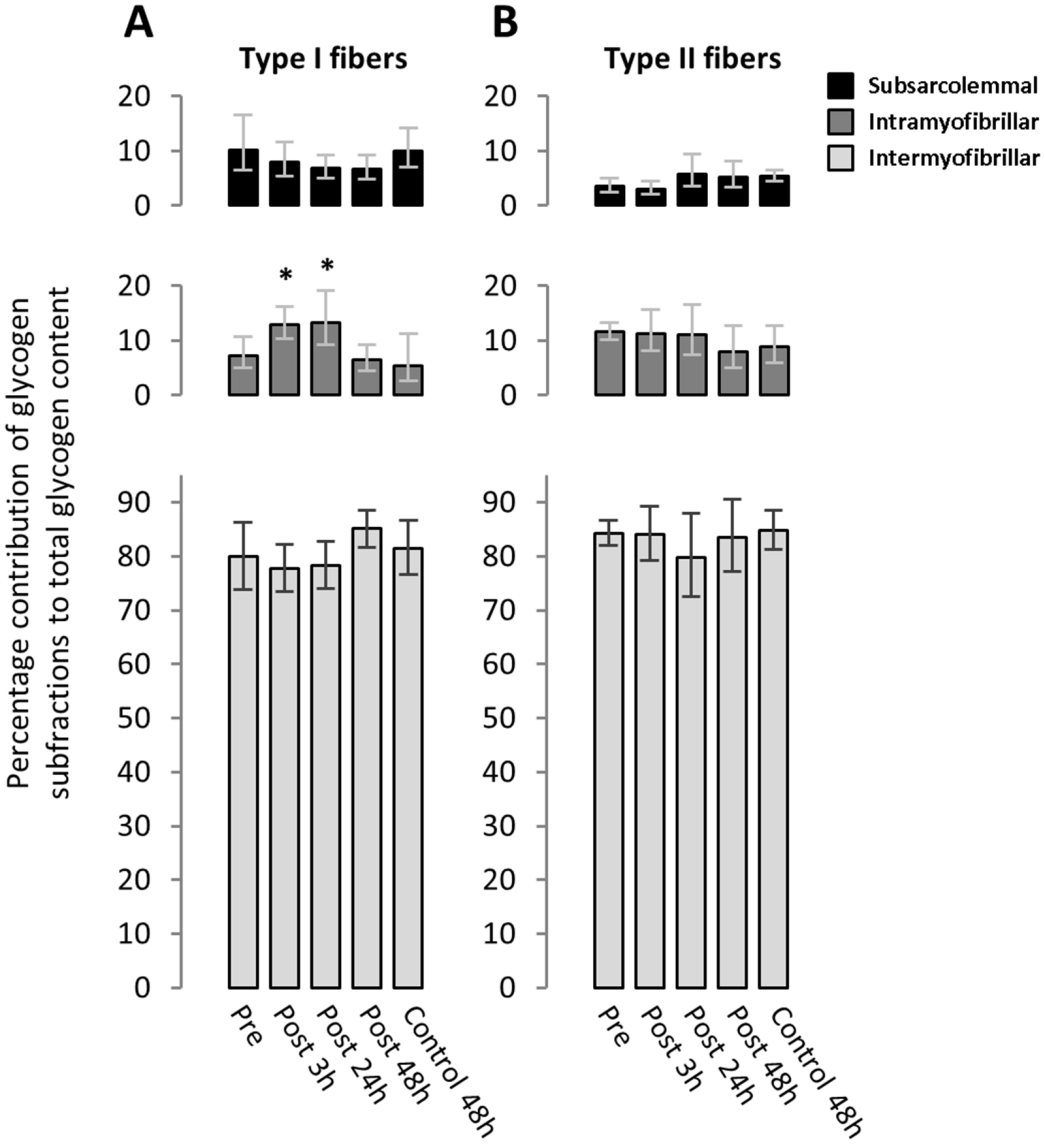
Increased relative contribution of intramyofibrillar glycogen to total glycogen of type I fibers after eccentric contractions. The percentage contribution of intermyofibrillar (light grey), intramyofibrillar (dark grey) and subsarcolemmal (black) glycogen to total glycogen content are shown for type I fibers (A) and type II fibers (B). *: different from Pre (P < 0.02, Kruskal-Wallis test). Values are geometric means and horizontal bars represent 95% confidence interval.

The enhanced storage of intramyofibrillar glycogen early during recovery from the eccentric contractions changed the relative distribution of glycogen within the fibers (Fig 3). In the type I fibers, the relative contribution of intramyofibrillar glycogen to total glycogen increased (P = 0.006, Kruskal-Wallis test) from a pre-exercise level of 7.3 (5.0–10.7) to 12.9 (10.3–16.2) and 13.3 (9.3–19.1) at post 3 h (P = 0.02, Kruskal-Wallis test) and at post 24 h (P = 0.01, Kruskal-Wallis test), respectively (Fig 3).

**Fig 4:**
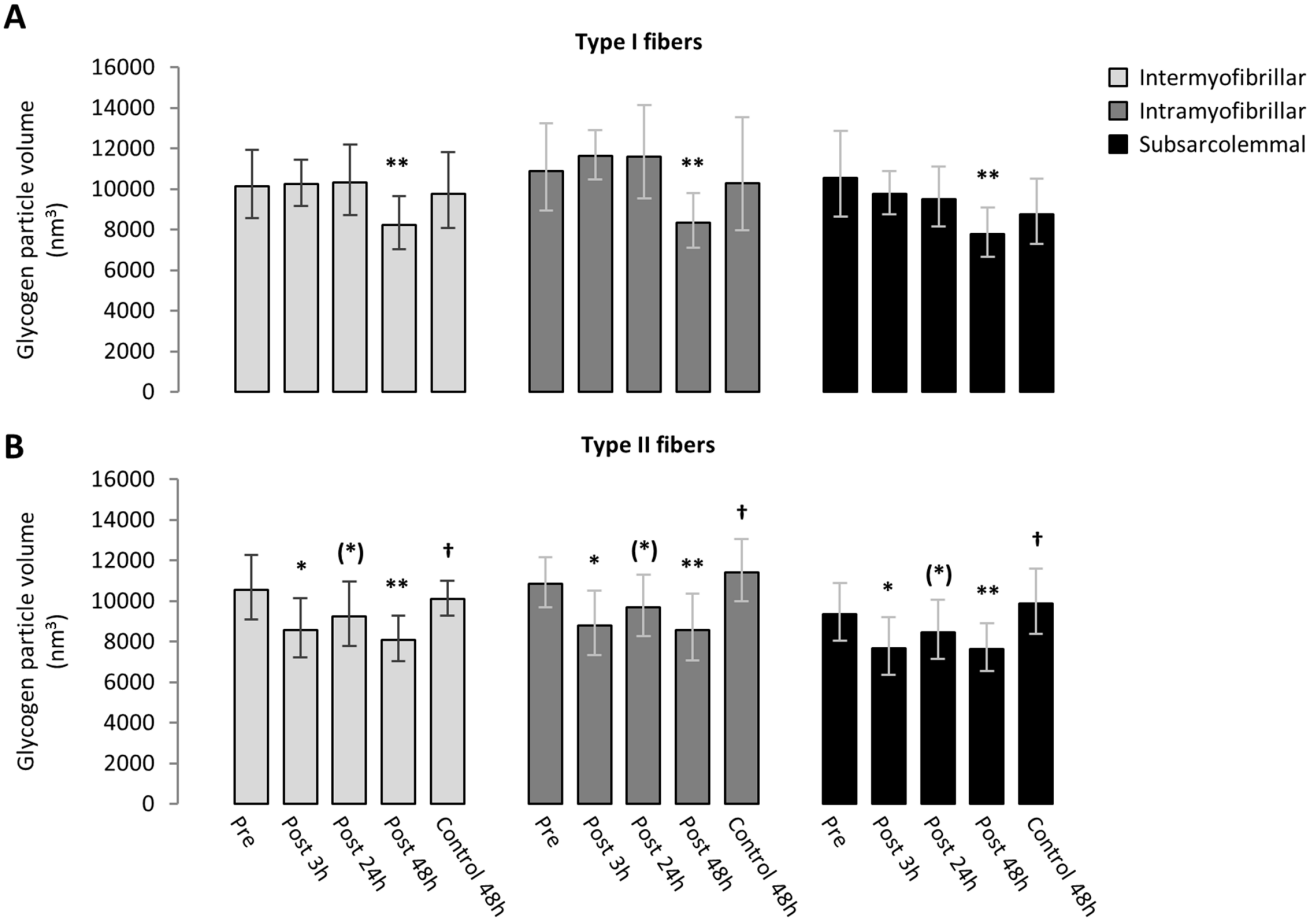
Changes in glycogen particle volume during recovery from eccentric contractions depend on fiber type and time course of recovery. The size of intermyofibrillar (light grey), intramyofibrillar (dark grey) and subsarcolemmal (black) glycogen particles are shown for type I fibers (A) and type II fibers (B). *: different from Pre (P = 0.02). (*): tendency to be different from Pre (P = 0.06). **: different from Pre (P < 0.001). †: different from Post 48h (P < 0.0001). Values are geometric means and horizontal bars represent 95% confidence interval.

**Fig 5:**
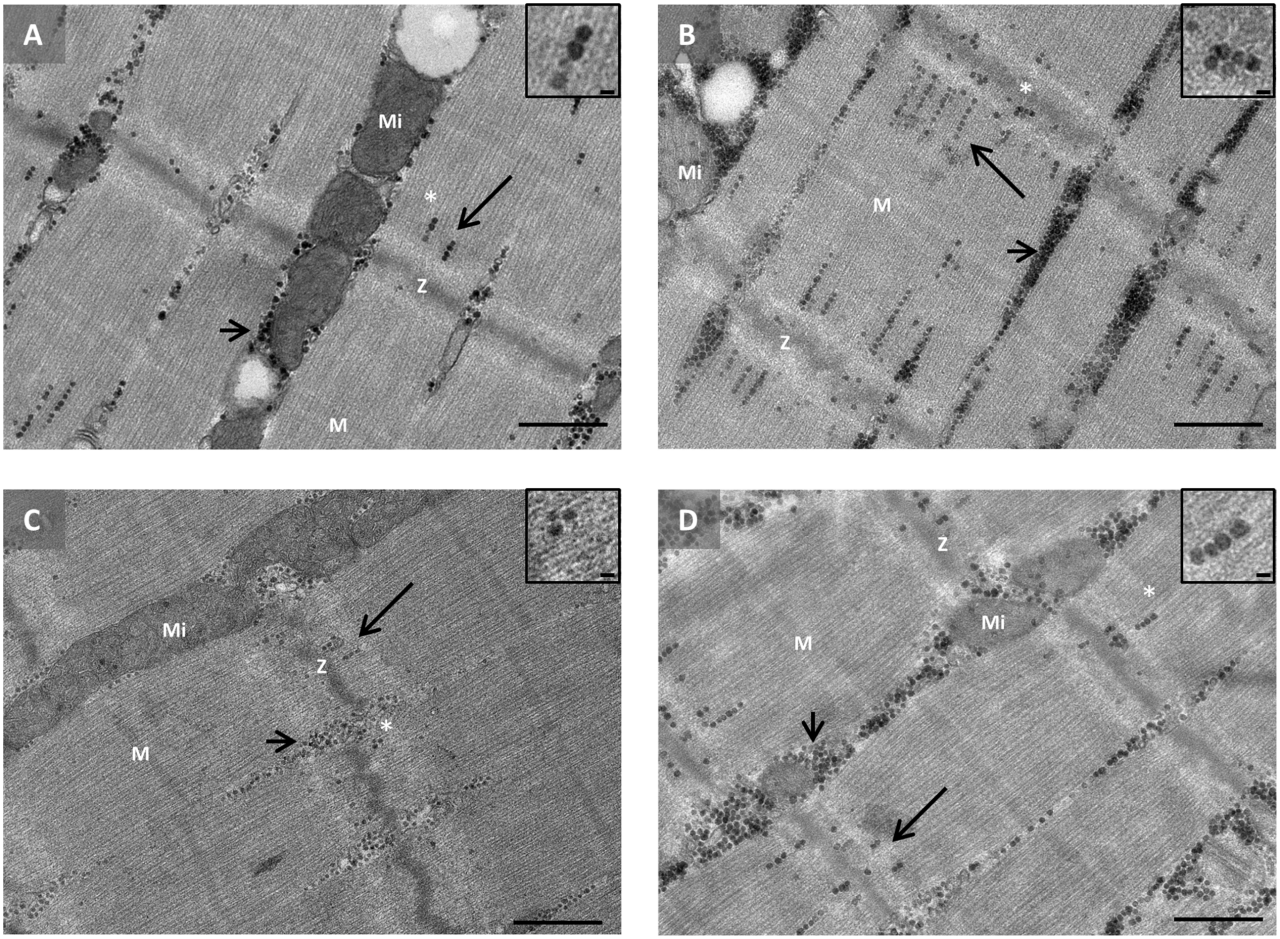
Representative transmission electron micrographs showing the enhanced storage of intramyofibrillar glycogen in type I fibers at 24 h post exercise (B) compared with pre exercise (A), and the lower content of intermyofibrillar and intramyofibrillar glycogen at 48 h post exercise (C) compared with the control leg (D). Glycogen particles are seen as black dots. Long arrows indicate intramyofibrillar glycogen and short arrows indicate intermyofibrillar glycogen. Mi, mitochondria. Z, Z-disk. M, M-band. In the upper left corner, selected glycogen particles are shown at a higher magnification (selected region indicated by asterisk). Scale bar: 0.5 μm and 30 nm in large images and inset boxes, respectively.

The average glycogen particle volume for each subcellular location of type I and II fibers during recovery from the eccentric exercise is presented in Fig 4. While, in type I fibers, the average glycogen particle volume did not change during early (3–24 h) recovery in any of the three subfractions of glycogen particles (Fig 4A), it was lowered by -21% (-26:-15) at post 48 h compared with the pre exercise value (Fig 4A). In type II fibers, on the other hand, the average glycogen particle volume was reduced by -16% (-27:-6), -10% (-18:01), and -20% (-27:-12) compared with pre exercise at post 3 h, 24 h and 28 h, respectively, and also lowered by -28% (-34:-21) compared with the control leg at 48 h. These changes in glycogen particle size during recovery from the eccentric contractions were not dependent on location in the type I fibers (P = 0.34) or the type II fibers (P = 0.99). Representative images are shown in Fig 5.

## Discussion

The main finding of the present study was that muscle-damaging eccentric exercise affected the subcellular distribution of glycogen content in a fiber type-dependent manner during post-exercise recovery. This effect was observed to be different in the early versus the later stage of post-exercise recovery.

With regard to the early event of post-exercise recovery (i.e. at 3 and 24 h post exercise) intramyofibrillar glycogen content (particles located within the myofibrils) was 150% higher compared with basal level, whereas intermyofibrillar and subsarcolemmal glycogen (particles located between the myofibrils or just beneath the surface membrane, respectively), did not change (see Fig 2). Furthermore, this higher level of intramyofibrillar glycogen early in the recovery period was only observed in fibers rich in mitochondria, a characteristic feature of type I fibers. Later in recovery (48 h post exercise) all three subfractions of glycogen (intramyofibrillar, intermyofibrillar and subsarcolemmal) in both fiber types were on average 35% lower compared with the control leg, although not lower than pre-exercise level (Fig 2). These findings suggest that two temporally distinct mechanisms influenced glycogen metabolism during recovery from muscle-damaging eccentric contractions. Furthermore, while the enhanced storage of intramyofibrillar glycogen content early during recovery could be ascribed a higher number of visible particles, the later decrease in glycogen was due to a decrease in the size of existing particles (see Fig 4).

### Enhanced storage of intramyofibrillar glycogen 3 and 24 hours after eccentric contractions

The enhanced storage of intramyofibrillar glycogen early during recovery was only observed in type I fibers (Fig 2E vs 2F) suggesting that the underlying mechanism is fiber type-dependent or that the exercise bout exerted differentials effects on the distinct fiber types. As for the fiber-type-specific mechanisms, translocation of glycogen synthase in response to exercise, occur to similar extent in both type I and type II fibers [30] indicating that this potential mechanism cannot explain the fiber type difference found in the present study. A more likely explanation is that the eccentric exercise protocol affected the two fiber types differently. Type I fibers have a slightly higher content of the insulin- and contraction-regulated glucose transporter isoform GLUT4 [31] and contraction-induced increase in glucose uptake in type I fibers is maximal immediately after contractions compared with a more delayed increase in glucose uptake in type II fibers [32]. Further, while contraction- and insulin-induced glucose uptake are additive in type I fibers, they seems not to be additive in type II fibers [32], which suggest that post-exercise carbohydrate supplementation in the present study likely favored glucose uptake of the type I fibers. However, potential differences between fiber types in contraction- and insulin-induced glucose uptake during early recovery after eccentric contractions, calls for further investigation.

In addition to differences in glucose uptake properties between the two fiber types, their susceptibility to muscle damage seems also to be different. In accordance, Brockett et al. [22] reported that fast-twitch motor units were more prone to muscle damage after eccentric contraction than slow-twitch motor units. Furthermore, Fridén et al. [20] showed that in some, but not all individuals, Z-disk streaming after eccentric exercise predominantly occurred in type II fibers. Consequently, the impaired glycogen synthesis of type II fibers compared with type I fibers in the present study could relate to impaired glucose uptake as induced by muscle damage [8, 9, 33]. On the other hand, assessment of Z-disc streaming, revealed only relatively small differences between fiber types (Fig 1). However, our ability to conclude on this, likely suffer from a combination of a limited sample size combined with very localized Z-disc-streaming. Future studies are therefore needed to investigate in more detail how eccentric contractions affect different fiber types not only at the level of z-disk streaming, but also at the level of sarcolemmal damage and extracellular matrix integrity (see [2]) and how this applies to fiber type- and subcellular localization-specific insulin- and contraction-mediated glucose uptake during early recovery. Yet, since the enhanced storage of intramyofibrillar glycogen occured only 3 and 24 h post exercise and only in the type I fibers, while the sarcomeric damage was observed throughout the immediate 48 h of post exercise recovery and in both type I and II fibers, no immediate association seems evident between sarcomere damage and ability to store intramyofibrillar glycogen.

The size of intramyofibrillar glycogen particles did not significantly change during early recovery (Fig 4B) indicating that the enhanced storaged of intramyofibrillar glycogen content is mediated by an increase in the number of glycogen particles. Here it is important to keep in mind that the currently available method, limits the detection threshold for glycogen particles to 10–12 nm. Consequently, the increased number of visible glycogen particles may rely on an increase in the size of small (< 10 nm) previously unrecognized particles and/or a translocation of particles from other locations. In support of this explanatory model, it has been shown that the fraction of glycogenin (the self-glucosylating protein backbone of glycogen) located within the myofibrils, does not change during the immediate 5 hours of recovery after glycogen-depleting exercise [34], indicating no translocation of particles. On the other hand, the regulation of glycogenin content may underlie other means than glycosylation, so this and the inherent effects on localization and activity of glycogenin in humans remain poorly understood [35]. The key enzyme in glycogen synthesis, glycogen synthase, has been shown to translocate from the intermyofibrillar region to the intramyofibrillar region in response to exercise [30]. This suggests that the enhanced storage of intramyofibrillar glycogen found early during recovery is mediated by a translocation of glycogen synthase to existing small glycogen particles (non-visible by the electron microscope) or just to a complex of glycogenin with a string of glucose residues. Intriguingly, an increase in small previously unrecognized particles rather than an equal increase in all particles is in line with glycogenin-overexpression studies showing no increase in glycogen content [36, 37], but an increase in number of small particles [36]. The implications of the role of the number of glycogen particles for muscle function remain to be elucidated. At present, there is no evidence for the other possible mechanism, i.e. translocation of glycogen particles. However theoretically, movement of glycogen particles along the cytoskeleton could be speculated to occur similarly to the movement of mitochondria [38] and glucose transporters [39].

Surprisingly, the high intramyofibrillar glycogen levels of type I fibers at 3 and 24 h post exercise, are matching the resting levels found in elite endurance trained athletes [40]. These high levels of intramyofibrillar glycogen found in endurance-trained athletes are likely to be a result of regular physical activity or high carbohydrate-intake (see below) rather than a long-term training-adaptation.

In this regard, it is important to note that the subjects of the present study were a part of a larger study [23], in which carbohydrate supplements were provided during the 48 h post-exercise recovery period investigated in the current study. Therefore, the high levels of intramyofibrillar glycogen found early during recovery could be an effect of carbohydrate loading in addition to the exercise performed. However, the intramyofibrillar glycogen level at 48 h after the exercise bout is not different from before the exercise bout and is actually lower than the control leg (Fig 2F) indicating that carbohydrate intake alone cannot sustain such a high intramyofibrillar glycogen level. In addition, it should also be noted that by defining the fibers as type I and II by having mitochondrial content within the highest or lowest tertile, respectively, the subjects did not provide with an equal number of fibers at all time-points. In order to address whether this bias has influenced the results, another fiber typing method was employed where the number of fibers from each subject was restricted to two fibers per time point and fibers with mitochondrial content close to the lower or upper tertile were also included. The results of these analysis revealed that the unequal number of fibers per subject were unlikely to have influenced the results (S1 Fig).

### Reduced glycogen content late in recovery from eccentric contractions

The late (i.e., 48 h post exercise) reduction in all subfractions of glycogen following the eccentric contractions is in accordance with previous findings of impaired glycogen resynthesis and sustained low glycogen levels in muscle homogenates following muscle-damaging eccentric contractions [4–7, 33]. These sustained low glycogen levels after eccentric exercise may occur in as consequences of lower GLUT4 content [7], increased glycogenolysis [26] and/or increased resting metabolism [41, 42].

Our observation that all subfractions of glycogen were equally affected was in discordance with our hypothesis that muscle-damaging eccentric contractions would primarily affect intramyofibrillar glycogen content. Importantly, with the limited sample size of only 5 subjects and the given intermyofiber and intersubject variation the difference between the late reduction in intramyofibrillar glycogen and intermyofibrillar or subsarcolemmal glycogen was 34 and 38%, respectively (95% confidence limit). With reference to previous observations of impaired resynthesis of intramyofibrillar glycogen 2 days after a muscle-damaging soccer match [18], this impairment is likely not mediated by eccentric contractions per se. In accordance, it has been previously found that intramyofibrillar glycogen is preferentially restored after glycogen depleting exercise [19] suggesting that a subcellular location-specific effect of eccentric contractions on glycogen synthesis is dependent on prior glycogen depletion with exercise. In addition, we found that the size of the glycogen particles decreased post 48 h as a consequence of muscle-damaging exercise. Whether this decrease is a result of a change in some of the proteins associated with the particles [43] following muscle damage remains to be investigated.

## Conclusion

The present study demonstrates that in the carbohydrate-supplemented state, a single exercise bout of eccentric contractions mediates an early (3–24 h post exercise) and substantial enhanced storage of glycogen of a subcellular hot spot within the myofibrils (intramyofibrillar glycogen) specifically in type I fibers. Furthermore, a late (48 h post exercise) reduction in glycogen content in all three defined subcellular locations, was observed, but in a fiber type independent manner. These findings suggest that eccentric contractions influences glycogen metabolism by two temporally separated and fiber type-specific as yet unknown mechanisms.

## Perspectives

The high levels of intramyofibrillar glycogen 3–24 h after the exercise bout observed in the present study may be important for muscle function and endurance performance. During prolonged high-intensity exercise, intramyofibrillar glycogen is preferentially depleted [19, 40] and this seems to be interrelated with impaired calcium release from sarcoplasmic reticulum[44] and fatigue development [26, 45]. In a randomized, controlled setting, it would therefore be relevant to delineate the underlying molecular mechanism, to investigate whether high levels of intramyofibrillar glycogen content is beneficial for muscle function and exercise performance and to resolve how it can be optimized.

## Supporting Information

S1 Fig Fiber type-dependent subcellular distribution of glycogen in skeletal muscle fibers during recovery from eccentric contractions as evaluated by two different fiber-typing methods. In fiber-typing method 1 (A-F) the fibers with mitochondrial content within the lowest and highest tertiles were defined as type I and II fibers, respectively, irrespective of subjects and time points. The fibers within the middle tertile were discarded. The two points of mitochondrial content that divided the fibers in three parts were 0.056 and 0.086 μm^3^ μm^-3^. Because of inter-biopsy variability in mitochondrial content of the fibers each subject contributed with unequal number of fibers per time point (listed below fig E-F). In fiber-typing method 2 (G-L), a more balanced number of fibers per subject was achieved by 1) allowing a maximum of only 2 fibers per subject per time point and 2) changing the cut-off values of mitochondrial content from below 0.056 to below 0.060 μm^3^ μm^-3^ for type II fibers and from above 0.086 to above 0.080 μm^3^ μm^-3^ for type I fibers. The results obtained by the two different fiber typing-methods were not meaningfully different. Bars represent geometric means and horizontal lines represent 95% confidence interval.
(TIF)
