# Supplementary figures and images for: Enhanced Glycogen Storage of a Subcellular Hot Spot in Human Skeletal Muscle during Early Recovery from Eccentric Contractions
Source: PLoS One. 2015 May 21;10(5):e0127808. doi: 10.1371/journal.pone.0127808 (PMC4440641; doi:10.1371/journal.pone.0127808)

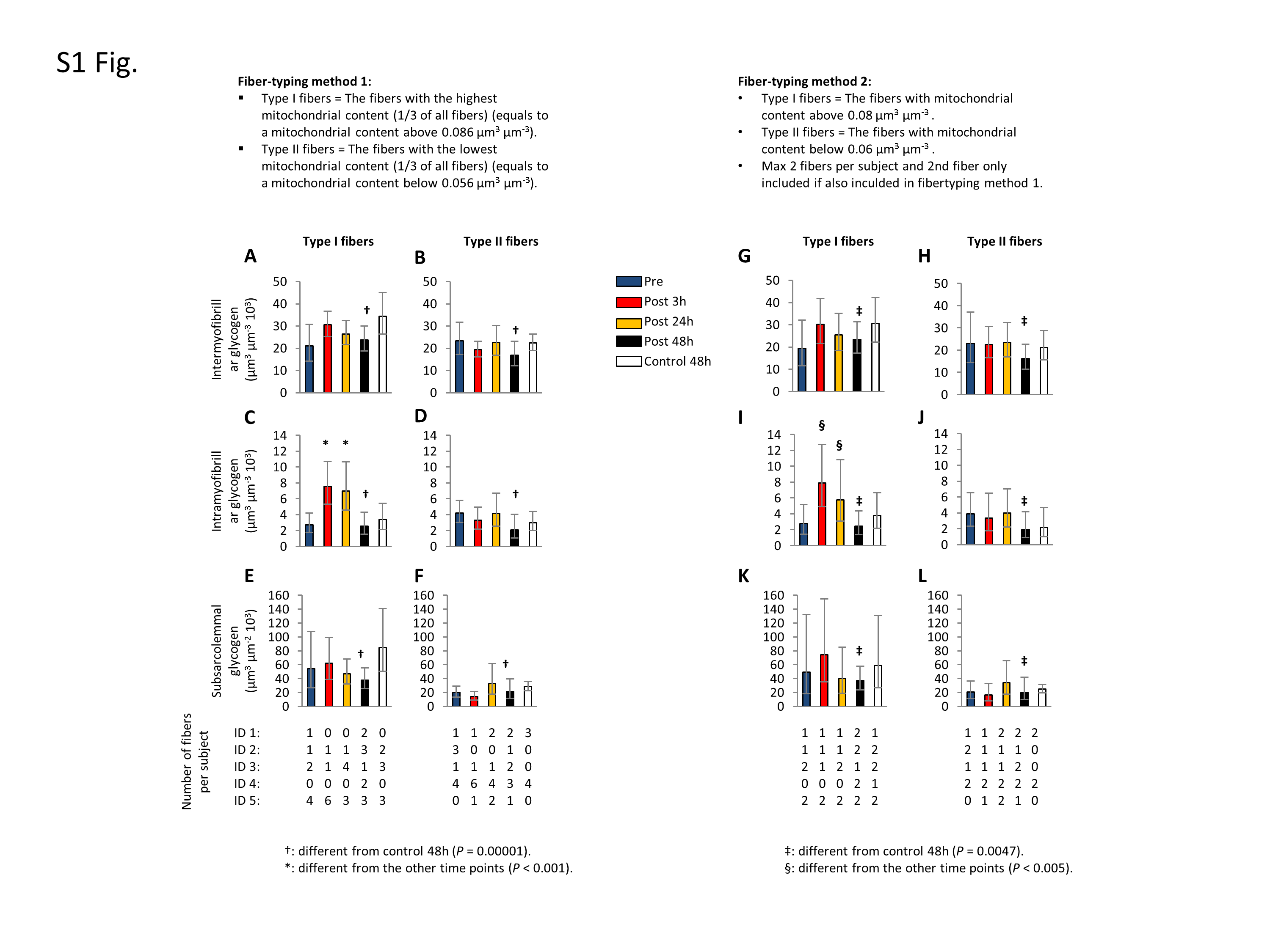

Supplement: S1 Fig — In fiber-typing method 1 (A-F) the fibers with mitochondrial content within the lowest and highest tertiles were defined as type I and II fibers, respectively, irrespective of subjects and time points. The fibers within the middle tertile were discarded. The two points of mitochondrial content that divided the fibers in three parts were 0.056 and 0.086 μm3 μm-3. Because of inter-biopsy variability in mitochondrial content of the fibers each subject contributed with unequal number of fibers per time point (listed below fig E-F). In fiber-typing method 2 (G-L), a more balanced number of fibers per subject was achieved by 1) allowing a maximum of only 2 fibers per subject per time point and 2) changing the cut-off values of mitochondrial content from below 0.056 to below 0.060 μm3 μm-3 for type II fibers and from above 0.086 to above 0.080 μm3 μm-3 for type I fibers. The results obtained by the two different fiber typing-methods were not meaningfully different. Bars represent geometric means and horizontal lines represent 95% confidence interval. (TIF) [file pone.0127808.s001.tif]
